# Effects of diet, habitual water intake and increased hydration on body fluid volumes and urinary analysis of renal fluid retention in healthy volunteers

**DOI:** 10.1007/s00394-020-02275-4

**Published:** 2020-05-19

**Authors:** Robert G. Hahn

**Affiliations:** 1 Research Unit, Södertälje Hospital, 152 86, Södertälje, Sweden; 2 Karolinska Institutet at Danderyds Hospital (KIDS), Solna, Sweden

**Keywords:** Urine analysis, Dehydration, Hydration status, Fluid retention

## Abstract

**Purpose:**

To increase our knowledge about the causes and physiological consequences of concentrated urine, the relevance of which in the general population is uncertain.

**Methods:**

Twenty healthy volunteers (mean age 42 years) recorded all intake of food and water for 2 weeks. During the 2nd week, they increased their daily consumption of water by 716 mL (32%). The volunteers delivered a 24-h and a morning urine sample for analysis of osmolality and creatinine during the first 4 days of both weeks, and a sample each time they voided on the other days. The water content of food and liquid was calculated and the body fluid volumes were measured by bioimpedance. Haemodynamic stability was assessed with the passive leg-raising test.

**Results:**

There was a curvilinear correlation between the daily intake of water and biomarkers measured in the 24-h collection of urine (coefficient of determination 0.37–0.70). Habitual low intake of water was associated with larger body fluid volumes. The increased fluid intake during the 2nd week was best reflected in the 24-h collection (−15 and −20% for the osmolality and creatinine, respectively, *P* < 0.002), while morning urine and body fluid volumes were unchanged. Increased fluid intake improved the haemodynamic stability in volunteers with a low intake of water (< median), but only in those who had minimally concentrated morning urine.

**Conclusions:**

The 24-h collection reflected recent intake of fluid, whereas the morning urine seemed to mirror long-term corrections of the fluid balance. Concentrated urine was associated with larger body fluid volumes.

## Introduction

Urine analysis of metabolic waste products is a tool for detecting dehydration in sports medicine [1–3]. Researchers have used comparisons of the degree of urine concentration in athletes with the decrease in their body weight during strenuous exercise to construct a urine colour scale that indicates the degree of dehydration [3, 4]. Eight such studies have even been used to construct a correlation curve between four pooled biomarkers of concentrated urine and known exercise-induced fluid loss [5].

Dehydration is also of interest in general medicine, and concentrated urine is associated with a high 30-day mortality in acute geriatric care [6], an increase in body weight after surgery [7], more complications after hip fracture surgery [7], and greater need for fluid to aid the circulation before surgery [9]. Dehydration changes the kinetics of crystalloid fluid given by intravenous infusion [10, 11] and probably increases the risk of having a postoperative elevation of plasma creatinine [12, 13]. However, concentrated urine is also common in the general population. Spot urine sampling showed the presence of renal water conservation to a degree corresponding to exercise-induced dehydration in 38% of 300 hospital workers, which is probably intentional due to the nature of their work [14].

Dehydration affects physical and mental capacity [15, 16]; therefore, the present study was undertaken to explore in greater detail the background underlying the high incidence of concentrated urine in healthy humans. The primary study hypothesis was that concentrated urine is due to low consumption of water and can be alleviated by increasing the intake of water. For this purpose, two biomarkers of concentrated urine were measured over a period of 2 weeks, where fluid intake was increased in the 2nd week (primary outcome measure). Registrations of body fluid volumes and haemodynamic measurements were also performed (secondary outcome measures). A second hypothesis was that the habitual or increased fluid intake changes the body fluid volumes and a third hypothesis was that a spot urine sample reflects the daily excretion of biomarkers or the urine volume, provided that a certain time interval has elapsed since the last meal.

## Materials and methods

During the third week of October 2016, 150 hospital workers volunteered for a screening study for concentrated urine. Twenty of these volunteers who presented with either very concentrated or very dilute urine were later asked to participate in a 2-week follow-up fluid balance study. The study was conducted according to the guidelines laid down in the Declaration of Helsinki and all procedures involving human subjects were approved by the Regional Ethics Committee of Stockholm (June 15, 2016, Dnr. 2016/826-31, Chairperson Hans Glaumann) and registered in an international database, https://www.isrctn.com, as identifier ISRCTN 12,215,472. Written and verbal consent was obtained from all participants in both the screening study and in the fluid balance study.

### Screening

The screening study was advertised on the local Internet system. All staff were invited. Exclusion criteria were professional sports activities and any disease that required daily medication or a special diet. Enrolment was stopped when 150 participants had been recruited. Written informed consent was obtained from each volunteer. The participants delivered a fresh 10 mL spot urine sample and a health status self-examination questionnaire at the Research Unit. They were not allowed to ingest any fluid within 2 h prior to voiding. Concentrated urine was assessed by immediately measuring the urine specific weight with a Clinitek Status^®^ Analyser (Siemens Healthcare Diagnostics).

### Fluid balance study

Twenty subjects from the screening study were invited to participate in a 2-week fluid balance study. The goal was to include only healthy subjects who had delivered very diluted or concentrated urine and to avoid emphasis on those presenting with average values. The chosen volunteers were interviewed for motivation and then informed about the study, both orally and in writing, and they finally signed a participation document. A health status examination was also performed including heart and lung auscultation before the study started.

The fluid balance study consisted of four periods:

- Days 1–4**:** Subjects were asked to drink as they usually do. Two samples were collected each day: a 24-h urine collection and a sample of the morning urine. The voided volume was recorded.
- Days 5–7: Subjects were asked to drink as they usually do. Urine was sampled every time the subject voided. The volume was not recorded.
- Days 8–11: Subjects were asked to drink 1.2 L more fluid than normal each day by adding one glass of water to each meal. Two samples were collected each day: a 24-h urine collection and a sample of the morning urine. The voided volume was recorded.
- Days 12–14**:** Subjects were asked to drink 1.2 L more fluid each day by adding one glass of water to each meal. Urine was sampled every time the subject voided. The volume was not recorded.

The study protocol is outlined in schematic form in Table 1.

**Table 1:** The scheme for the fluid balance study

Study days	Normal fluid intake		Increased fluid intake	
	Days 1–4	Days 5–7	Days 8–11	Days 12–14
Urine sampling	Morning and 24-h collection	Each void	Morning and 24-h collection	Each void
Diet protocol	Yes	Yes	Yes	Yes

The urine osmolality and the urinary creatinine concentration were used as an index of the renal conservation of water. The osmolality and the creatinine concentration were measured within 36 h with an Advanced 2020 osmometer (Molek AB, Sweden) and Cobas 8000 analyser (Roche Diagnostics, Basel, Switzerland), respectively, at the certified clinical laboratory at Karolinska University Hospital in Stockholm. The CV was 3% for osmolality and 5% for creatinine (at 6 mmol/L). The rationale for using these biomarkers is that solutes and creatinine are excreted at a fairly stable rate regardless of fluctuations in urine flow rate. Urine creatinine is even used routinely in laboratory science worldwide to correct urinary concentrations for dilution effects.

The fluid balance study was performed with three to six volunteers per month between November 2016 and March 2017. The volunteers were never told if they had concentrated or diluted urine, and the investigator was blinded to the results until all interactions with the volunteers had been completed.

### Food record

The participants ate and drank freely, but were asked to keep a weighed food record for every day of the 2 weeks that the fluid balance study lasted. They weighed all ingested foodstuffs on a scale and recorded the type of food and the time of the meal in a protocol. The nutritional content was calculated by a dietician using the Dietist Net software (Kostoch Näringsdata, Bromma, Sweden). This program (available in Swedish, English, Norwegian and Danish at https://www.kostdata.se) is based on data on the average nutritional content of foodstuffs, according to the Swedish National Food Agency and the US Department of Agriculture. The results were expressed as the numbers of calories and the amounts of protein, fat, carbohydrates, fibre, alcohol, and liquid, and the total amount of ingested water per meal and the total for each day. The content of specialised products, such as protein-enriched food and smoothies, was entered separately, based on the declaration of content on the product packaging.

### Body fluid volumes and fluid responsiveness

Assessment of body weight, body fluid volumes and haemodynamics was performed on three occasions: in the morning on the day before the study started and on day 7 and 14. Testing was conducted after 2 h had passed since any intake of food or water.

The body weight was measured on an electronic scale (Vetek AB, Väddö, Sweden).

Multifrequency bioelectrical impedance, determined using a Xitron 4000B Spectrum Analyser (Xitron Technologies Inc., San Diego, CA), was used to estimate the extracellular, intracellular and total body fluid water volumes (ECV, ICV, and TBW, respectively) [17, 18].

The haemodynamic stability during the “passive leg-raising test” is of interest in fluid balance disorders [19, 20] and was tested using a non-invasive haemodynamic monitor (Nexfin, BMEYE, Amsterdam, NL) [21]. A large increase in cardiac output when the legs are lifted (> 10%) is called “fluid responsiveness” and indicates that tissue perfusion is not optimal, which is a sign of dehydration [20]. During surgery, “fluid responsiveness” serves as an indication for the administration of intravenous infusion of fluid. In the present study, the passive leg-raising test was a functional haemodynamic test used to indicate underhydration. With an optimal fluid balance, the increase in cardiac output about 1 min after having the legs raised from semi-recumbent position to a 45° angle is smaller than 10%, or even becomes reduced. Technical details about how to perform this test are found elsewhere [22]. Lean body mass was estimated by using anthropometric equations [23].

### Statistics

Group data are presented as the mean and standard deviation (SD). Differences between groups were studied by the one-way and analysis of variance (ANOVA), whereas the paired *t* test was applied to changes during the study. Analysis of group differences combined with changes over time were studied by two-way ANOVA. Correlations between water intake (independent variable) and the urinary biomarkers (dependent variable) were studied by simple and multiple linear regression analysis, where *r*^2^  is the coefficient of determination, which is the proportion of the variance of *y* that can be explained by *x*. These analyses were conducted using StatView SE + Graphics v.1.02 software (Abacus Concepts, NJ), and *P* < 0.05 was accepted as statistically significant.

Based on a previous report of concentrated and dilute urine of volunteers [14], the study was powered to detect a change in urine osmolality of 200 mosmol/kg when water intake was increased (standardised difference 1.5), with a power of 95% at the *P* < 0.05 level (the software used was GPower version 3.1.9.2).

## Results

### Demographics

The 20 volunteers (16 women and 4 men) who agreed to participate in the fluid balance study were aged 42 ± 11 years (range 23–62). The food record from one volunteer was lost and could not be reliably reconstructed. All others completed the study, which comprised 1778 meals and 1123 measurements of urine osmolality and creatinine.

### Overall correlations

The intake of liquid accounted for 76 ± 10% of the total water consumption (correlation *r*^2^ = 0.91). A similar fraction of the ingested water, 68 ± 15%, was excreted as urine (*r*^2^ = 0.84).

**Fig. 1:**
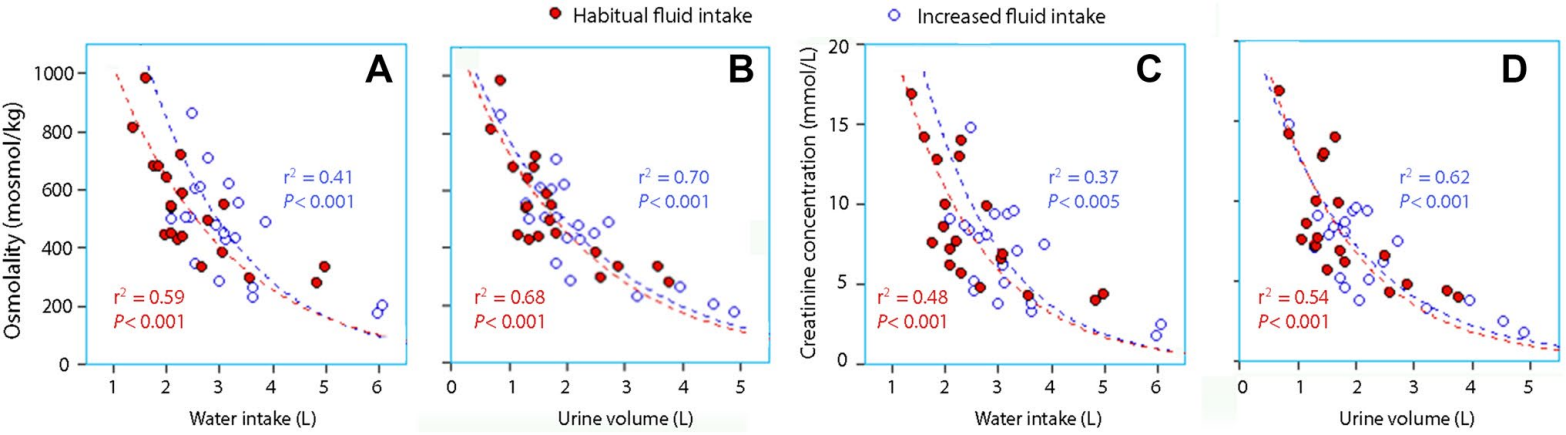
Daily intake of water (**a**, **c)** and the urinary volume (**b**, **d)** versus the urine osmolality (**a**, **b)** and creatinine concentration (**b**, **c**) in 24-h collections of urine. Each point represents the mean for one volunteer during habitual (days 1–4) or increased fluid intake (days 5–8). Logarithm transformation of the data on the y-axis was used for the regression analysis

**Fig. 2:**
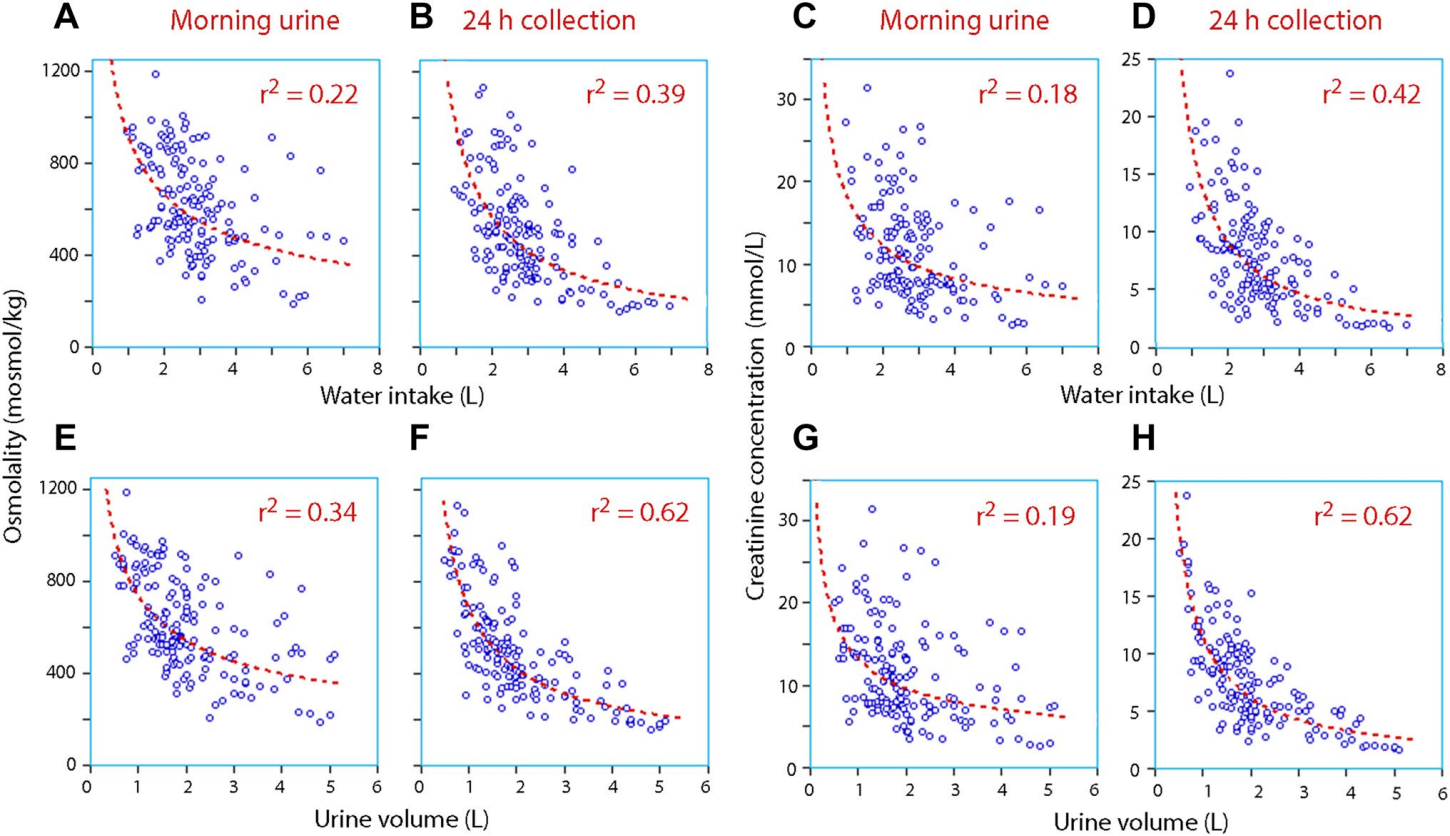
Daily intake of water (**a**–**d**) and the urine volume (**e**–**h**) versus the urine osmolality (**a**, **b**, **e**, **f**) and creatinine concentration (**c**, **d**, **g**, **h**) in the morning and in 24-h collections of urine. Each subplot represents 4 days of habitual and 4 days of increased fluid intake in 20 volunteers (160 data points); therefore, each shows the sum of intra- and inter-subject variability. Logarithm transformation of the Y-axis data was used for the regression analysis

**Fig. 3:**
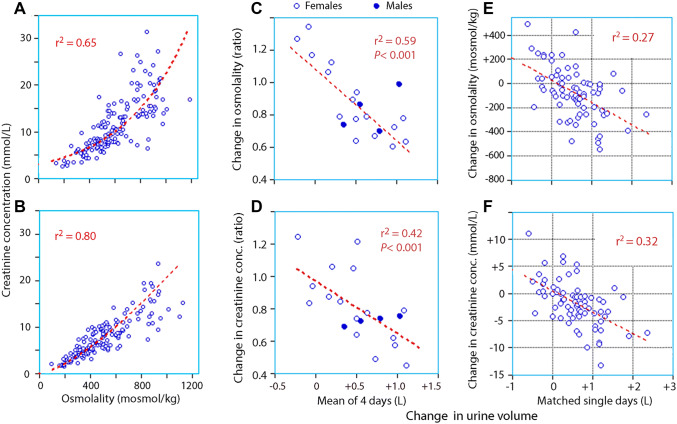
The osmolality versus the creatinine concentration in (**a**) the morning urine and (**b)** the 24-h collection. (**c**–**f**): Relationships between the changes in urine osmolality and creatinine concentration in the 24-h collection between the period with increased and normal fluid intake versus the simultaneous change in excreted urine volume. Subplots (**c**) and (**d**) compare the mean values for the biomarkers for days 8–11/days 1–4, whereas subplots (**e**) and (**f**) give the absolute changes on matched individual days (day 8 cf. day 1, day 9 cf. day 2, etc.)

The water intake and the urine volume correlated strongly with the logarithm-transformed osmolality and the creatinine concentration in the 24-h collection of urine, while the biomarkers measuring the morning urine correlated less well. Figure 1 illustrates these relationships based on the participant mean values. Figure 2 shows the raw data for the 8 days of measurement and highlights the sum of intra- and inter-subject variability. The osmolality and the creatinine concentration also correlated with each other, both when measured in the morning urine (Fig. 3a) and in the 24-h collection (Fig. 3b).

Young volunteers (< 40 years) had more concentrated urine than the older ones (> 40 years). For example, the osmolality in the 24-h collection of urine during the first 4 days was 614 ± 194 mosmol/kg in the young and 449 ± 133 mosmol/kg in the older volunteers (*P* < 0.02) and the creatinine concentration showed 10.4 ± 4.0 vs. 6.8 ± 2.6 mmol/L (*P* < 0.03). The same trend was found in the morning urine. The young volunteers had a tendency to ingest less water than the older, 31 ± 9 vs. 39 ± 18 mL/kg body weight (*P* = 0.20), although their body weights were the same, 73 ± 11 vs. 72 ± 11 kg (*P* = 0.78).

Males had higher urinary creatinine concentrations than females, both in the morning urine (16.1 ± 7.0 vs. 10. 5 ± 4.0 mmol/L; *P* < 0.045) and 24-h urine collection (12.4 ± 1.7 vs. 7.6 ± 3.5 mmol/L; *P* < 0.02), but the urine osmolality did not differ significantly between males and females.

### Increased fluid intake

During the 2nd week, the intake of water was 32% higher (716 ± 425 mL) and ingestion of oral liquid was 47% higher (+ 764 ± 440 mL) when compared to the first week. The intake of calories, protein, fat, carbohydrates and fibre did not change significantly (Table 2).

**Table 2:** Intake of food and water and the measurements of concentrated urine. Participant mean values over two 4-day periods (*N* = 20)

	Normal fluid intake days 1–4	Increased fluid intake days 8–11	*P* value
**Food record**			
Energy (kcal)	1921 ± 399	2072 ± 666	0.08
Protein (g)	83.1 ± 17.3	87.8 ± 23.4	0.31
Fat (g)	84.6 ± 22.4	93.6 ± 26.1	0.13
Carbohydrates (g)	177 ± 87	195 ± 63	0.24
Fibre (g)	14.9 ± 5.3	14.4 ± 5.2	0.61
Oral liquids (mL)	1877 ± 837	2641 ± 970	< 0.001
Water, total (mL)^a^	2564 ± 981	3280 ± 1068	< 0.001
Water/energy (ratio)	1.41 ± 0.59	1.69 ± 0.66	< 0.001
**Urine analysis**			
Morning urine			
Osmolality (mosmol/kg)	621 ± 160	596 ± 175	0.45
Creatinine (mmol/L)	11.9 ± 5.1	11.1 ± 4.3	0.31
24-h collection			
Osmolality (mosmol/kg)	536 ± 186	455 ± 178	< 0.01
Creatinine (mosmol/kg)	8.7 ± 3.8	6.9 ± 3.2	< 0.001
Volume (mL)	1771 ± 874	2322 ± 1094	< 0.001

Data are the mean ± SD. The paired *t* test was used to compare the two time periods

^a^Sum of the water content of both ingested liquid and food

The hydration period caused a 35% increase in urine volume, but the osmolality and the creatinine concentration of the morning urine did not change significantly (−3%). By contrast, the 24-h collection showed a decrease in osmolality by 15% (*P* < 0.002) and a decrease in creatinine by 20% (*P* < 0.001, Table 2). These changes were quite similar for the young (< 40 years) and old (> 40 years) volunteers, but the differences in urinary creatinine between males and females became smaller and non-significant.

The increase in urine volume induced by the increased hydration correlated with simultaneous changes in the biomarkers, but only in the 24-h collection (Fig. 3c–f).

### Low versus high habitual fluid intake

The volunteers were separated into two groups depending on whether their intake of water during days 1–4 was below or above the median for all participants.

In those with a low habitual intake of water, increased fluid intake reduced the osmolality and creatinine in the 24-h collection by 17% (*P* < 0.01 and *P* < 0.02, respectively), whereas no changes were observed in the morning urine.

In volunteers with a high habitual intake of water, the osmolality of the 24-h collection was reduced by 13% (*P* = 0.14) and creatinine by 23% (*P* < 0.003). The morning urine showed only small numerical reductions of these biomarkers (Table 3).

**Table 3:** Urine analysis and daily fluid consumption in volunteers with a low or high water intake during days 1–4. Participant mean values over two 4-day periods were compared

Urine analysis	Low water intake (< median)		High water intake (> median)	
	Days 1–4	Days 8–11	Days 1–4	Days 8–11
**Morning urine**				
Osmolality (mosmol/kg)	697 ± 135	703 ± 150	526 ± 148	477 ± 117
Creatinine (mmol/L)	13.0 ± 3.8	12.9 ± 4.3	10.7 ± 6.1	9.1 ± 3.9
**24-h collection**				
Osmolality (mosmol/kg)	632 ± 170	523 ± 219**	430 ± 147	380 ± 165
Creatinine (mmol/L)	9.9 ± 3.5	8.0 ± 3.1*	7.4 ± 3.9	5.7 ± 2.9*
Urine volume (mL)	1208 ± 296	1719 ± 418***	2337 ± 856	2865 ± 1235**
**Oral intake of fluid**				
Water, total (mL)^a^	2003 ± 459	2634 ± 312***	3188 ± 1,049	3999 ± 1164***
Oral liquids (mL)	1418 ± 317	2007 ± 316***	2387 ± 952	3347 ± 971***

The paired *t* test was used to compare days 1–4 with days 8–11 in each subgroup

*P < 0.05, **P < 0.01, ***P < 0.001

^a^Sum of the water content of both ingested liquid and food

Two-way ANOVA applied to data from all volunteers confirmed that increased fluid intake reduced the osmolality of the 24-h collection (*P* < 0.012) and that the change tended to be greater in those with low habitual consumption of water (interaction effect *P* = 0.14). Similarly, there was an overall decrease in creatinine concentration (*P* < 0.001), but the degree of change was similar in the low and high water intake subgroups (interaction effect *P* = 0.49). No statistically significant differences or changes were disclosed when two-way ANOVA was applied to the morning urine.

Increased water intake did not significantly change the body fluid volumes in any of the groups (Table 4).

**Table 4:** Daily nutrient consumption, body weight, body fluid volumes and arterial blood pressures in 20 volunteers with low (< median) or high (> median) spontaneous intake of water

	Low water intake	High water intake	*P* value
**Food record days 1–14**			
Calories/b.w. (kcal/kg/day)	24.8 ± 3.8	26.7 ± 4.3	0.29
Protein/b.w. (g/kg/day)	1.06 ± 0.20	1.22 ± 0.19	0.10
Fat/b.w. (g/kg/day)	1.13 ± 0.27	1.18 ± 0.30	0.70
Carbohydrates/b.w. (g/kg/day)	2.33 ± 0.81	2.54 ± 0.59	0.52
Fibre/b.w. (g/kg/day)	0.18 ± 0.07	0.24 ± 0.08	0.08
Oral liquids, total (mL/kg/day)	25.9 ± 6.3	38.3 ± 15.0	< 0.03
**Body mass**			
Body weight (kg), day 1	70.5 ± 11.4	75.0 ± 10.1	0.36
Body weight (kg), day 7	70.1 ± 11.6	74.6 ± 10.1	0.36
Body weight (kg), day 14	70.0 ± 11.4	74.7 ± 10.4	0.35
Height (cm)	166 ± 9	173 ± 7	< 0.05
Body mass index (kg/m^2^), Day 1	25.8 ± 4.5	25.1 ± 3.7	0.71
Lean body mass (%), Day 1^a^	48.2 ± 6.1	53.8 ± 6.2	0.06
**Body fluid volumes**			
ECV / b.w. (L/kg, %)			
Day 1	21.7 ± 2.2	21.6 ± 1.8	0.93
Day 7	22.0 ± 1.8	21.7 ± 1.6	0.73
Day 14	22.1 ± 1.8	21.6 ± 2.0	0.58
ICV / b.w. (L/kg, %)			
Day 1	28.6 ± 3.3	28.8 ± 6.1	0.93
Day 7	28.3 ± 2.9	28.5 ± 6.3	0.90
Day 14	28.4 ± 2.8	29.2 ± 5.6	0.71
TBW/b.w. (L/kg, %)			
Day 1	50.6 ± 5.2	50.4 ± 7.7	0.94
Day 7	50.2 ± 4.5	51.0 ± 7.7	0.80
Day 14	50.6 ± 4.2	50.8 ± 7.2	0.96
**Arterial pressures** (mmHg)			
Day 1 systolic	137 ± 24	136 ± 24	0.90
Diastolic	78 ± 12	78 ± 13	0.91
Day 7 systolic	119 ± 16	124 ± 14	0.54
Diastolic	69 ± 8	76 ± 8	0.09
Day 14 Systolic	133 ± 27	133 ± 27	0.54
Diastolic	80 ± 13	77 ± 14	0.09

Data are the mean ± SD of ten volunteers in each group. One-way ANOVA was used for statistics

*b.w* body weight, *ECF* extracellular fluid, fluid volume, *ICF* intracellular fluid, *TBW* total body water

^a^Estimated by using Boer’s formula [23]

### Body fluid volumes and fluid responsiveness

**Fig. 4:**
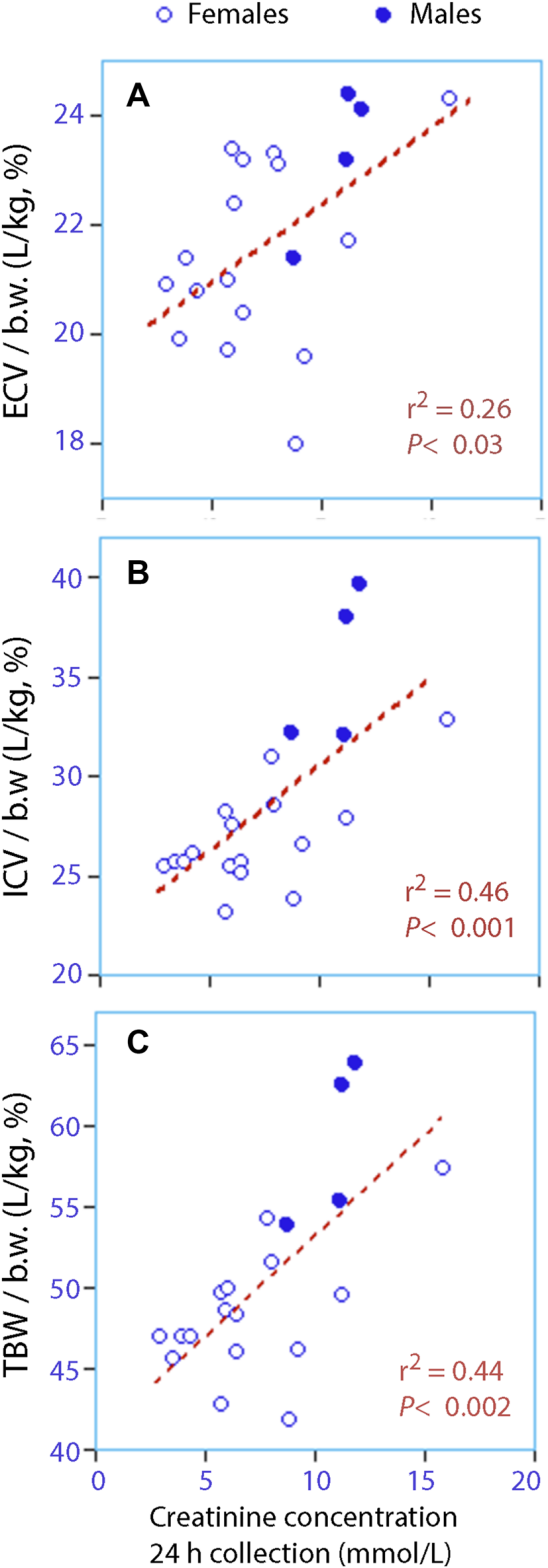
Relationships between the creatinine concentration in the 24-h collections of urine and **a** the extracellular fluid volume (ECV), **b** the intracellular fluid volume (ICV), and **c** the total body water (TBW) per kilo body weight (b.w.). Each point is the mean of all measurements for each individual participant, i.e., eight collections of biomarkers and three measurements of body fluid volumes

**Fig. 5:**
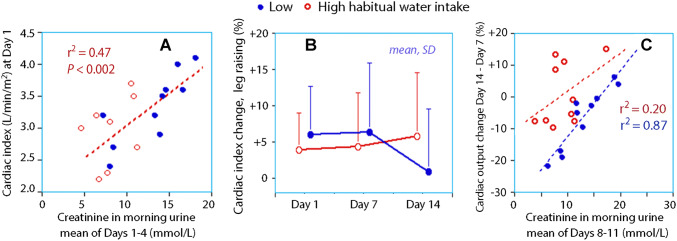
Haemodynamics. (**a**) Correlation between cardiac index and concentrated urine at baseline. **b** The cardiac index response to passive leg-raising depending on fluid intake; responses close to zero indicate haemodynamic stability. **c** Difference in cardiac index response between day 14 and day 7. More negative values imply a greater the effect of increased fluid intake (multiple linear regression; fluid intake group *P* < 0.002 and creatinine *P* < 0.001). One extreme outlier was omitted from panel **a**

The body fluid volumes were larger (Fig. 4) and cardiac index was higher (Fig. 5a) when the biomarkers indicated fluid retention.

Five volunteers showed fluid responsiveness during the first week, but only one did so at the end of the second week (from 25 to 10%). Increased fluid intake tended to reduce the haemodynamic response to the passive leg-raising test in those with low habitual intake of water (Fig. 5b, paired *t* test *P* = 0.053). Those with the greatest improvement had not yet developed concentrated morning urine (Fig. 5c).

### Spot samples

Urinary osmolality and creatinine showed higher values in spot samples (taken at any time of the day) from volunteers having a low habitual intake of water when compared to those having a high habitual intake of water (*P* < 0.01 and *P* < 0.03, respectively, Table 5).

**Table 5:** Spot urine analyses during the period of normal diet (days 5–7) and increased water intake (days 12–14)

Spot sample analysis	Low habitual water intake		High habitual water intake	
	Days 5–7	Days 12–14	Days 5–7	Days 12–14
Osmolality (mosmol/kg)	728 ± 182	513 ± 141	451 ± 176	405 ± 137
Creatinine (mmol/L)	11.6 ± 3.8	8.7 ± 4.2	7.8 ± 4.6	7.0 ± 3.4

Data are the mean ± SD of the mean values for each volunteer

Osmolality and creatinine were lower on days 12–14 vs. 5–7 by *P* < 0.001 and *P* < 0.03, respectively (paired *t* test)

Two-way ANOVA confirmed that the subject mean osmolality was reduced when the intake of water was increased (*P* < 0.001); this change was most pronounced among those with low habitual intake of water (interaction effect *P* < 0.013). Urine creatinine was also lower during the period of increased water intake (*P* < 0.03) with a trend for greater change among volunteers with a low habitual intake of water (*P* = 0.13).

**Fig. 6:**
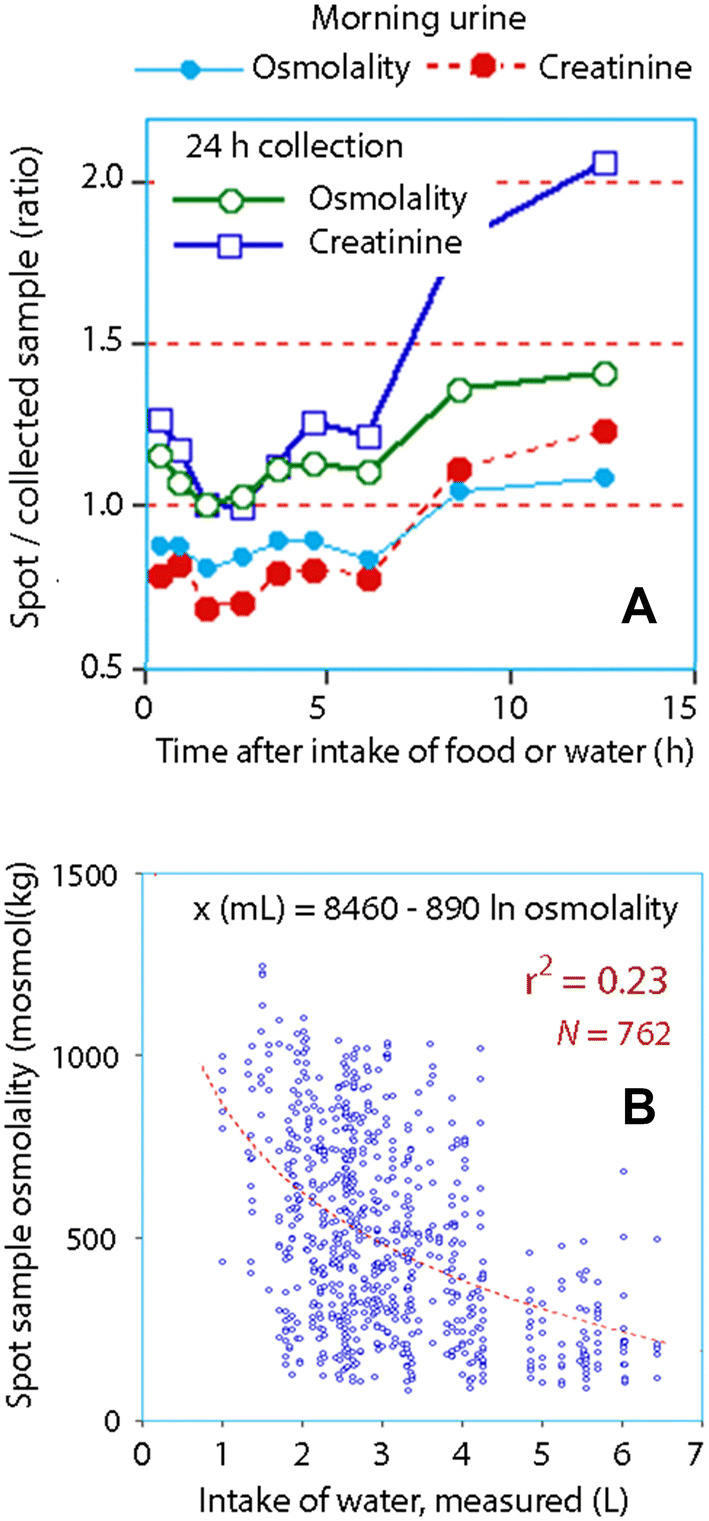
**a** Deviations of urinary spot samples (mean) from the means obtained during the first 4 days of each week weeks depending on the time after feeding or water intake; **b** any spot sample compared to the 24-h intake of water during the same day. The regression equation differed from the measured water intake by 629 mL (median)

Spot samples offered the most accurate predictions of the 24-h collections of biomarkers between 1 and 3 h after water intake/meal (close to 100%, *N* = 376), but the SD of 225 mosmol/kg for osmolality and 4.3 mmol/L for creatinine illustrate that the variability was great (Fig. 6a). Clinically useful predictions of the daily water intake from single spot samples could only be obtained at the extreme ends of Fig. 6b. For example, the positive predictive value of urine osmolality of > 600 mosmol/kg to indicate a daily intake of water < 1.7 L rather than > 3.0 L was 85% and the negative predictive value was 82%.

## Discussion

Measurements of metabolic waste products in the urine are easy to perform and are potentially useful tools for assessing the degree of body hydration [24]. This type of testing has been evaluated for exercise-induced dehydration in sports medicine [1–5], and they also seem to be relevant to hospital care [6–13]. The validity and importance of urine analysis as a surrogate measure of the fluid balance in the general population is still unclear, but the issue has recently caught interest [14, 24–28].

The present results confirm that concentrated urine is due to low consumption of water, but the answer to the second part of the primary hypothesis is mixed. Analysing the two biomarkers in a 24-h collection of urine reflected the recent intake of water and the excreted urine volume in a curvilinear fashion, and better than the morning urine did. The 24-h collections of urine also provided information about sudden changes in the intake of water, which was deliberately induced in the beginning of the second study week.

In contrast, the morning urine seemed to reflect a long-term adaptation of the fluid balance. This interpretation is based on the fact that both biomarkers correlated reasonably well with the fluid intake during the period of free water consumption, but hardly changed at all when the water intake was increased. Hence, short-term changes in water intake had no effect on the biomarkers, particularly not when the habitual intake of water was low, showing that elevated levels of biomarkers in the morning urine require more than 7 days to decrease in response to an increase in water intake by 32%.

The second hypothesis was refuted, as the body weight and the body fluid volumes showed no systematic changes in response to changes in water consumption. These findings suggest that long-term between-subject variations in fluid intake are responsible for the differences in body water shown in Fig. 4.

Johnson et al*.* [26] did find adjustment of the urinary biomarkers in the morning urine during a 4-day period in subjects with a low habitual intake of water, but this occurred in response to a three times greater increase in the water consumption than the one used here, from 1.6 to 3.6 L per day. Interestingly, the body weight still did not change. Stookey et al*.* reported an increase in body weight of 0.5 kg, but only after 4 weeks of increased water intake amounting to 1 L/day [27]. Following a 3-day fluid restriction to 1 L/day in volunteers with normal fluid intake, an additional 1.5 L/day was needed to regain normal urine colour, which is another index of concentrated urine, within 24 h [28]. Thus, a quite large change in water intake is needed to make the kidneys change from conserving to excreting water.

The biomarkers in the morning urine correlated best with the haemodynamic measurements (Fig. 5). The subgroup in which an increased intake of water improved the haemodynamic stability on the leg-raising test were the volunteers with low habitual intake of water who also had an inappropriately weak renal conservation of water (Fig. 5c). Hence, good matching between water intake and urine concentration seems to be needed to ensure full haemodynamic stability. Some subjects with a low habitual intake of water could perhaps have developed renal fluid retention only after some time, or were unable to do so effectively.

The measurements of body fluid volumes illustrate the effectiveness of the renal conservation of water. Use of the term “intracellular dehydration” for chronic low intake of water might even be questioned, because physiological compromises maintained, and even overcompensated, the body fluid volumes in most cases. Therefore, concentrated urine in the general population should be separated from the concentration of urine that occurs during acute exercise-induced dehydration, where ranges of urinary biomarkers correspond to specified losses of body weight [5].

The third hypothesis in the present study dealt with spot sampling; however, the usefulness of spot urine sampling to detect body hydration has been questioned [29]. The answer is that a spot urine sample does reflect the daily excretion of biomarkers, but with poor precision. The average difference between the measurements of biomarkers in spot samples and 24-h collections of urine were negligible between 1 and 3 h after ingestion of food or water (Fig. 6a). However, the errors associated with this estimation were large and fairly constant at all times after a meal, which can probably be explained by differences in eating patterns, meal compositions and rates of absorption from the gut. Another issue was whether spot urine sampling could be used to indicate the daily intake of water. In this study, meaningful predictions from a single spot sample could only be made to distinguish very low from very high consumption of water (Fig. 6b).

Hypohydration can arise either due to volume depletion, in which the ECV is lost, or to intracellular dehydration, which occurs due to sweating, evaporation, or low intake of water [30]. Hypohydration in exercising athletes is most safely diagnosed based on a rise in serum osmolality to > 300 mosmol/kg [1–4], but only marginal differences, if any, are found in serum osmolality between subjects with diluted and concentrated urine in the general population [14, 24, 26, 27]. Johnson et al*.* found a minor rise in plasma vasopressin, which acts to increase the TBW, in subjects with a low intake of water; this increase could explain why the urine became concentrated [31]. Conversely, increasing the daily consumption of water is known to decrease the vasopressin concentration [27]. Increased intake of liquid clearly contributed more than food did to differences in the total consumption of water (Table 3). Non-significant trends did suggest that volunteers also tended to ingest more foodstuffs when they drank more or were asked to do so, which has also been found by others [32].

Limitations include the downsides of self-reported fluid intake, where underreporting of unhealthy foodstuffs appears to be common [33]. No validation was made regarding the food reports, such as by using the Goldberg cutoff [34], but the volunteers were hospital staff (mostly nurses) who are well trained in following a protocol and reporting. However, not all of them increased their water consumptions during the 2nd week as much as advised. Both women and men were studied, which increased the variance of some parameters. Men and women ingested almost identical volumes of water, but their intake of foodstuff was similar only if corrected for body weight. As expected, males had more body water [35] and higher urinary creatinine concentrations [1–5, 14], probably due to their larger muscle mass.

The variance of the water consumption was also increased by the selection of participants from the screening study, which intended to compare healthy subjects who presented with very diluted or concentrated urine. Therefore, water consumption showed more extreme values than expected from population studies, although the average intake (2.5 L/day) was normal [36]. Spot samples were collected whenever the volunteers experienced an urge to void, while other authors have performed measurements at fixed times during the day [25, 28]. No control group was enlisted that maintained the habitual fluid and food intake throughout the 2-week period. Concentration of the urine might be a mechanism that maintains haemodynamic stability in humans with a low habitual intake of water, but the conclusions based on haemodynamics are underpowered, and a larger study is needed to confirm them. Our data indicated that ICV is much smaller than the commonly cited 40%, but the ranges in body fluid ratios shown in Fig. 4 have also been reported elsewhere [37–39].

In conclusion, a 2-week study in volunteers outlined inter-correlations between water intake and urine volume, and between two biomarkers of concentrated urine (urine osmolality and creatinine). Although low intake of water is apparently the key mechanism for the development of concentrated urine in healthy humans, the measured biomarkers correlated more strongly with the urine volume than they did with the intake of water. Spot urine samples best represented the urine collections when performed between 1 and 3 h after a meal, but could only distinguish between extreme deviations from normal fluid intake. A 24-h collection mirrored recent fluid balance events, whereas the morning urine seemed to reflect corrections of the body fluid volumes due to long-term (> 1 week) changes in habitual fluid intakes. The kidneys overcompensated chronically low fluid intakes and thereby expanded the body fluid volumes, while increased daily intake of water by 32% did not significantly increase the body fluid volumes or the body weight during a 7-day study period. Hence, concentrated urine in healthy humans is associated with low fluid consumption and normal or increased body fluid volumes, which is a condition that should be clearly separated from urine concentration due to exercise-induced dehydration. Studies are needed to find out if habitually low fluid intake or volume depletion is responsible for the associations between in-hospital morbidity and concentrated urine that have been published.
